# Abstract from an Interesting Paper on the Therapy of Various Forms of Light and Radio-Therapy

**Published:** 1903-05

**Authors:** 


### Reviews of Dental Literature.

Abstract from an Interesting Paper on the Therapy of Various Forms of Light and Radio-Therapy. By James MacFarlane Winfield, M.D.[1]

After careful search through the literature of radio-therapy it is safe to assume that over ninety per cent. of the cases of lupus treated by this method are cured. Only two cases have come under my personal observation; both were cured after twelve exposures, and have remained well for nearly a year.

The results in lupus erythematosus do not appear to be as good as those treated by the Finsen light.

Judging from the reports, radio-therapy has a beneficial and curative effect in about seventy-five per cent. of epithelioma, including rodent ulcer. My experience is about the same.

---

[1] Lecturer on Dermatology and Chief of Clinic at the Long Island College Hospital, etc.

At present I have under treatment an exceedingly interesting case of epithelioma, in a man eighty-one years of age. The cancer involved nearly the whole of the under lip. The universal opinion of the surgeons consulted was that it was an inoperable case, because of his age and the existence of a severe and far advanced Bright's disease.

The malignant growth had been aggravated by the application of caustic pastes so that when the ray was first applied the lip was a foul-smelling, suppurative mass. After the third application the discharge ceased. The ray has been applied twenty-six times, the average duration of sittings twelve minutes, and now the disease is practically cured.

The results in carcinoma, especially post-operative, are sufficiently good to warrant the following up of the surgical measure by the X-ray, and from the reports of careful observers radio-therapy should be employed in all cases of inoperable carcinoma.

During the past year I have used the ray in six cases of cancer of the breast, two primary and four post-operative. The first two received, respectively, four and eight exposures of ten minutes' duration, extending over a period of three months; in the one receiving four treatments the size of the tumor remained stationary, while previously it had grown rapidly.

In the other case the tumor began to diminish in size after three exposures; when she was last seen it was at least one-half smaller than before treatment. As these patients were hard to control, they were finally lost sight of.

In the four post-operative cases one died from exhaustion after only a few exposures to the ray; in one the skin and cicatrix were thickly studded with new growths; after twelve exposures the small tumors had entirely disappeared and the large ones were greatly diminished in size. The patient is still under observation, and although the ray has not been applied for nearly four months the malignant process does not seem to be making any headway.

The third case of inoperable carcinoma is interesting because it shows microscopically that the X-ray inhibits the growth of the carcinomatous cells. Six weeks after operation the patient was referred to me for treatment because of a rapidly growing tumor situated just outside of the cicatrix. The growth was hard and showed unmistakable signs of malignancy; decreased after eight treatments; then, for unavoidable reasons, the exposures were suspended for nearly three weeks. When the patient again presented herself it was found that the tumor had nearly doubled in size, and the overlying skin was inflamed (not, however, an X-ray dermatitis); surgical procedure was advised, and a second operation was immediately done; the growth was found to consist of broken-down material, and scattered through the adjacent fascia and deeper structures were numerous hard nodules; everything that appeared suspicious was removed and sent to the pathologist without any comments regarding the case or reference to the ray treatment; he reported that undoubtedly the tissues were carcinomatous, but they showed evidences of having undergone some peculiar change, which had stopped the cell growth. A similar observation regarding the power of X-ray over carcinomatous cells has recently been made by Mr. Stephen Mayou, an English physician. My patient is still under observation and treatment, and so far there are no signs of recurrence.

Considerable discussion is now going on regarding the beneficial effect of radio-therapy upon pelvic and abdominal malignant neoplasms; some claim that the growth of these tumors is stopped, and many times they disappear altogether.

While on this subject it is well to notice that the X-ray is capable of and does cause absorption, and conservative men have suggested that the use of a powerful ray might produce metastasis of the malignant process; this is a reasonable surmise, and before we advise or use radio-therapy in deep-seated or inoperable malignant disease the case should be thoroughly understood and all evidences carefully weighed; then if the results are grave we can feel assured that our patient has received the best that medical science can offer, even to the last resort, and the stigma of quackery is removed from a method that is of undoubted value.

Very few authoritative reports are obtainable regarding radio-therapy in the treatment of sarcoma, but from recent observations by Coley it appears that the ray has an inhibitory effect upon this form of cancer.

All agree that it lessens, and many times absolutely relieves, pain; this alone would be sufficient to warrant the continuance of this procedure in sarcoma.

I have used the ray in two cases, one of the jaw and the other of the glands of the neck, but neither derived any benefit except relief from pain.—*Brooklyn Medical Journal.*

